# The impact of fremanezumab on medication overuse in patients with chronic migraine: subgroup analysis of the HALO CM study

**DOI:** 10.1186/s10194-020-01173-8

**Published:** 2020-09-21

**Authors:** Stephen D. Silberstein, Joshua M. Cohen, Michael J. Seminerio, Ronghua Yang, Sait Ashina, Zaza Katsarava

**Affiliations:** 1 Jefferson Headache Center, Thomas Jefferson University, 900 Walnut Street, Second Floor, Philadelphia, PA 19107 USA; 2 Teva Branded Pharmaceutical Products R&D, Inc., West Chester, PA USA; 3 BIDMC Comprehensive Headache Center, Beth Israel Deaconess Medical Center, Harvard Medical School, Boston, MA USA; 4 Evangelical Hospital Unna, Unna, Germany; 5 Department of Neurology, University of Duisburg-Essen, Essen, Germany; 6 EVEX Medical Corporation, Tbilisi, Georgia; 7 IM Sechenov First Moscow State Medical University (Sechenov University), Moscow, Russian Federation

**Keywords:** Fremanezumab, Chronic migraine, Medication overuse

## Abstract

**Background:**

We evaluated the efficacy of fremanezumab, a fully humanized monoclonal antibody that selectively targets calcitonin gene-related peptide, in patients with chronic migraine (CM) with and without medication overuse (MO).

**Methods:**

In a 12-week, phase 3 trial, patients with CM were randomized to fremanezumab quarterly (675 mg/placebo/placebo), monthly (675 mg/225 mg/225 mg), or placebo. Post hoc analyses assessed the impact of fremanezumab in patients with and without MO (monthly use of acute headache medication ≥15 days, migraine-specific acute medication ≥10 days, or combination medication ≥10 days) on efficacy outcomes, including headache days of at least moderate severity (HDs), and six-item Headache Impact Test (HIT-6) and Migraine-Specific Quality of Life (MSQoL) questionnaire scores.

**Results:**

Of 1130 patients enrolled, 587 (51.9%) had baseline MO. Fremanezumab reduced placebo-adjusted least-squares mean (95% confidence interval) monthly HDs (− 2.2 [− 3.1 to − 1.2] and − 2.7 [− 3.7 to − 1.8]; *P* < 0.0001) in patients with MO and without MO (quarterly − 1.4 [− 2.3 to − 0.5], *P* = 0.0026; monthly − 1.4 [− 2.3 to − 0.6], *P* = 0.0017). Significantly more fremanezumab-treated patients had ≥ 50% reduction in HDs versus placebo, regardless of baseline MO (with: quarterly 70/201 [34.8%], monthly 78/198 [39.4%] vs placebo 26/188 [13.8%]; without: quarterly 71/174 [40.8%], monthly 75/177 [42.4%] vs placebo 41/183 [22.4%]). Fremanezumab improved HIT-6 and MSQoL scores. Significantly more fremanezumab-treated patients reverted to no MO (quarterly 111/201 [55.2%], monthly 120/198 [60.6%]) versus placebo (87/188 [46.3%]).

**Conclusions:**

Fremanezumab is effective for prevention of migraine in patients with CM, regardless of MO, and demonstrated a benefit over placebo in reducing MO.

**Trial registration:**

ClinicalTrials.gov NCT02621931 (HALO CM), registered December 12, 2012.

## Background

Individuals with chronic migraine (CM) frequently use acute headache medications, including triptans, ergot derivatives, opioids, and simple and combination analgesics, which can result in medication overuse (MO) [1, 2] and lead to greater disability and further reduced quality of life [1–5]. Preventive migraine therapy is recommended in patients with failure or overuse of acute medication, frequent attacks (≥4 headache days per month), headaches that interfere with daily routines despite acute treatment, or adverse events associated with acute treatments [3, 6]. Despite the potential benefits, preventive therapy is often underutilized [7, 8, 9], and persistence with treatment is often poor due to lack of efficacy or intolerable side effects [10–12]. New preventive therapies may improve patient lives with superior treatment efficacy and tolerability compared with previously available migraine preventive therapies [12, 13].

Fremanezumab, a fully humanized monoclonal antibody (IgG2∆a) that selectively targets calcitonin gene-related peptide (CGRP), is approved in the United States and the European Union for the preventive treatment of migraine in adults [14, 15]. The 12-week, double-blind, placebo-controlled, phase 3 HALO CM trial demonstrated that subcutaneous administration of fremanezumab significantly reduced headache days of at least moderate severity in patients with CM [16]. To understand the impact of fremanezumab in patients with MO, data from patients with CM with and without MO were assessed post hoc to examine the reduction of headache and migraine days and acute headache medication use. Outcomes were also examined in patients who either did or did not revert from MO at baseline to no MO during the study.

## Methods

### Standard protocol approvals, registrations, and patient consents

The study (NCT02621931; https://clinicaltrials.gov/ct2/show/NCT02621931) was conducted in accordance with the International Conference for Harmonisation guidelines for Good Clinical Practice, the Declaration of Helsinki, and relevant national and local regulations, and it followed the established study protocol [16]. The protocol was approved by relevant ethics committees and institutional review boards, and written informed consent was obtained from each patient prior to performing any study procedures or assessments [16].

### Study design, patients, and treatments

A description of the randomized, double-blind, placebo-controlled, parallel-group phase 3 HALO CM study (NCT02621931), which included a screening visit, 28-day pretreatment period, 12-week treatment period, and final evaluation (week 12), has been published previously [16].

Briefly, adults aged 18 to 70 with a history of migraine (according to the International Classification of Headache Disorders, third edition [ICHD-3] beta criteria [17]) for ≥12 months prior to screening and prospectively confirmed CM (headache on ≥15 days and ≥8 days fulfilling ICHD-3 beta criteria for migraine, probable migraine, or use of triptan or ergot medications) during the 28-day pretreatment baseline period were eligible to participate [16]. Patients who used opioids (including codeine) or barbiturates on >4 days per month were excluded from the trial. Additional inclusion and exclusion criteria are provided in a Table in Additional file 1.

Eligible patients were randomized 1:1:1 to receive subcutaneous injections of either fremanezumab quarterly (675 mg of fremanezumab at baseline and placebo at weeks 4 and 8), fremanezumab monthly (675 mg of fremanezumab at baseline and 225 mg at weeks 4 and 8), or placebo (matching placebo at baseline and at weeks 4 and 8) [16].

### Outcomes

The primary endpoint of the study, mean change from baseline (28-day pretreatment period) in the monthly average number of headache days of at least moderate severity during the 12-week treatment period, has been previously described [16]. Herein, post hoc analyses were conducted to assess the impact of fremanezumab in patients with and without MO. Patients were grouped based on the presence or absence of baseline MO, which was defined as use of acute headache medication on ≥15 days, migraine-specific acute medication on ≥10 days, or combination medications for headache on ≥10 days during the 28-day pretreatment period [17].

The following outcomes were assessed in patients with and without MO based on mean change from baseline (28-day pretreatment period) in the: monthly average number of headache days of at least moderate severity during the 12-week treatment period; monthly average number of migraine days during the 12-week treatment period; and monthly average number of days of any acute headache medication use. Also assessed was the proportion of patients with a ≥ 50% reduction from baseline (28-day pretreatment period) in the monthly average number of headache days of at least moderate severity during the 12-week treatment period; mean change from baseline (day 0) in scores on the six-item Headache Impact Test (HIT-6; scores range from 36 to 78, with higher scores indicating greater impact of headache on functional status and well-being) [18] at 4 weeks after the last dose of study drug; mean change from baseline (day 0) in domain scores on the Migraine-Specific Quality of Life (MSQoL) questionnaire (domains assessed: role function–restrictive [RFR; seven items on how migraines limit daily activities], role function–preventive [RFP; four items on how migraines prevent these activities], emotional function [EF; three items on the emotional effects of migraines]; scores range from 0 to 100, with higher scores indicating better health-related quality of life) [19] at 4 weeks after the last dose of study drug; and mean change from baseline (day 0) in scores on the nine-item Patient Health Questionnaire (PHQ-9; scores range from 0 to 27, with depression severity categorized as no or minimal [0 to 4], mild [5 to 9], moderate [10 to 14], moderately severe [15 to 19], and severe [20 to 27]) [20] at 4 weeks after the last dose of study drug.

Additionally, patients with MO at baseline were assessed for reversion to no MO, where reversion was defined as no longer meeting criteria for MO over the 12-week period, or continued MO during the 12-week treatment period. Outcomes assessed in these subgroups included those previously described.

### Statistical analyses

The post hoc analyses reported here were performed on subgroups of patients in the full analysis set (FAS) population: randomized patients who received ≥1 dose of study drug and had ≥10 days of post-baseline efficacy assessments on the primary endpoint (i.e., a daily headache diary) [16]. The subgroups included patients with MO at baseline versus without MO at baseline; and patients who did revert from MO at baseline to no MO during the study versus patients who did not revert from MO at baseline to no MO during the study. Least-square (LS) mean changes from baseline were evaluated using an analysis of covariance with treatment, sex, region, and baseline preventive migraine medication use as fixed effects, and baseline values and years since onset of migraines as covariates. The Cochran-Mantel-Haenszel test was used to analyze treatment differences between proportions of patients, with baseline preventive medication use as the stratification variable. Patients who prematurely discontinued from the study were considered as non-responders for overall analysis. For subgroups defined by treatment outcomes (i.e., patients who did or did not revert from MO at baseline to no MO during the study), data were summarized using descriptive statistics.

## Results

### Study population

A total of 1130 patients with CM were randomized to receive fremanezumab quarterly (*n* = 376), fremanezumab monthly (*n* = 379), or placebo (*n* = 375). The criteria for CM with MO at baseline were met by 201 (53.6%), 198 (52.8%), and 188 (50.7%) of the evaluable patients in the fremanezumab quarterly, fremanezumab monthly, and placebo groups, respectively. The demographics and clinical characteristics of patients in each subgroup (with MO and without MO) differed from each other in several ways, most notably age, years since initial diagnosis, current preventive medication use, current use of triptans or ergots, prior topiramate use, prior onabotulinumtoxinA use, headache days of at least moderate severity, migraine days, and days of acute headache medication use, although statistical significance of differences between subgroups in individual characteristics was not tested (Table 1).

**Table 1:** Demographics and baseline characteristics with and without MO^a^

	CM With MO			CM Without MO		
	Fremanezumab			Fremanezumab		
	Quarterly (*n* = 201)	Monthly (*n* = 198)	Placebo (*n* = 188)	Quarterly (*n* = 174)	Monthly (*n* = 177)	Placebo (*n* = 183)
Age, mean (SD), y	44.6 (11.6)	44.8 (10.9)	45.0 (10.8)	39.0 (12.6)	36.2 (11.5)	37.7 (12.1)
Sex, female, n (%)	183 (91)	173 (87)	168 (89)	147 (84)	154 (87)	158 (86)
BMI, mean (SD), kg/m^2^	26.4 (5.3)	26.4 (5.0)	26.0 (5.0)	26.9 (5.5)	26.7 (5.3)	26.9 (5.1)
Disease history						
Years since initial migraine diagnosis, mean (SD)	21.0 (12.7)	22.8 (12.3)	23.2 (13.8)	18.3 (12.9)	17.1 (11.0)	16.7 (11.0)
Current preventive medication use, n (%)	47 (23.4)	54 (27.3)	38 (20.2)	30 (17.2)	31 (17.5)	39 (21.3)
Current use of triptans or ergots, n (%)	141 (70.1)	138 (69.7)	128 (68.1)	67 (38.5)	49 (27.7)	64 (35.0)
Prior topiramate use, n (%)	65 (32.3)	72 (36.4)	77 (41.0)	41 (23.6)	43 (24.3)	40 (21.9)
Prior onabotulinumtoxinA use, n (%)	38 (18.9)	34 (17.2)	32 (17.0)	28 (16.1)	16 (9.0)	17 (9.3)
Disease characteristics during the 28-day pretreatment period						
Number of headache days of at least moderate severity,^b^ mean (SD)	15.5 (5.0)	14.9 (5.4)	14.8 (5.4)	10.5 (4.8)	10.5 (5.3)	11.7 (5.8)
Number of migraine days,^c^ mean (SD)	17.2 (4.6)	17.5 (4.9)	17.3 (5.0)	15.0 (4.9)	14.3 (5.0)	15.4 (5.1)
Number of days of acute medication use, mean (SD)	18.1 (4.0)	18.4 (4.5)	18.2 (4.4)	7.4 (4.4)	7.1 (4.5)	7.7 (4.5)
HIT-6 score, mean (SD)	64.5 (5.1)	65.3 (4.4)	63.8 (5.2)	64.0 (4.3)	63.9 (4.3)	64.3 (4.4)
PHQ-9 score, mean (SD)	4.3 (5.5)	5.9 (6.7)	3.7 (5.7)	4.1 (5.6)	4.0 (5.6)	4.0 (5.6)
MSQoL domain scores						
RFR, mean (SD)	49.1 (18.9)	46.8 (19.7)	49.7 (20.5)	48.2 (18.3)	49.7 (18.7)	48.8 (19.2)
RFP, mean (SD)	67.5 (21.1)	63.2 (23.5)	67.5 (22.9)	67.1 (20.4)	68.7 (20.6)	67.4 (21.7)
EF, mean (SD)	58.4 (26.2)	54.3 (27.1)	58.0 (27.5)	55.8 (26.9)	60.4 (25.1)	57.7 (25.4)

*BMI* Body mass index, *CM* Chronic migraine, *EF* Emotional function, *HIT-6* Six-item Headache Impact Test, *MO* Medication overuse, *MSQoL* Migraine-Specific Quality of Life, *RFP* Role function−preventive, *RFR* Role function−restrictive, *SD* Standard deviation

^a^MO was defined as use of acute headache medication on ≥ 15 days, migraine-specific acute medication on ≥ 10 days, or combination medication for headache on ≥ 10 days during the 28-day pretreatment period. ^b^A headache day of at least moderate severity was defined as a calendar day in which headache pain lasted at least 4 consecutive hours and had a peak severity of at least a moderate level, or a day in which acute migraine-specific medication (triptan or ergot) was used to treat a headache of any severity or duration. ^c^A migraine day was defined as a calendar day in which headache pain lasted at least 4 consecutive hours and met criteria for migraine (with or without aura) or probable migraine (subtype in which only one migraine criterion is absent), or a day in which acute migraine-specific medication (triptan or ergot) was used to treat a headache of any duration.

### Monthly average number of headache days of at least moderate severity with and without MO

**Fig. 1:**
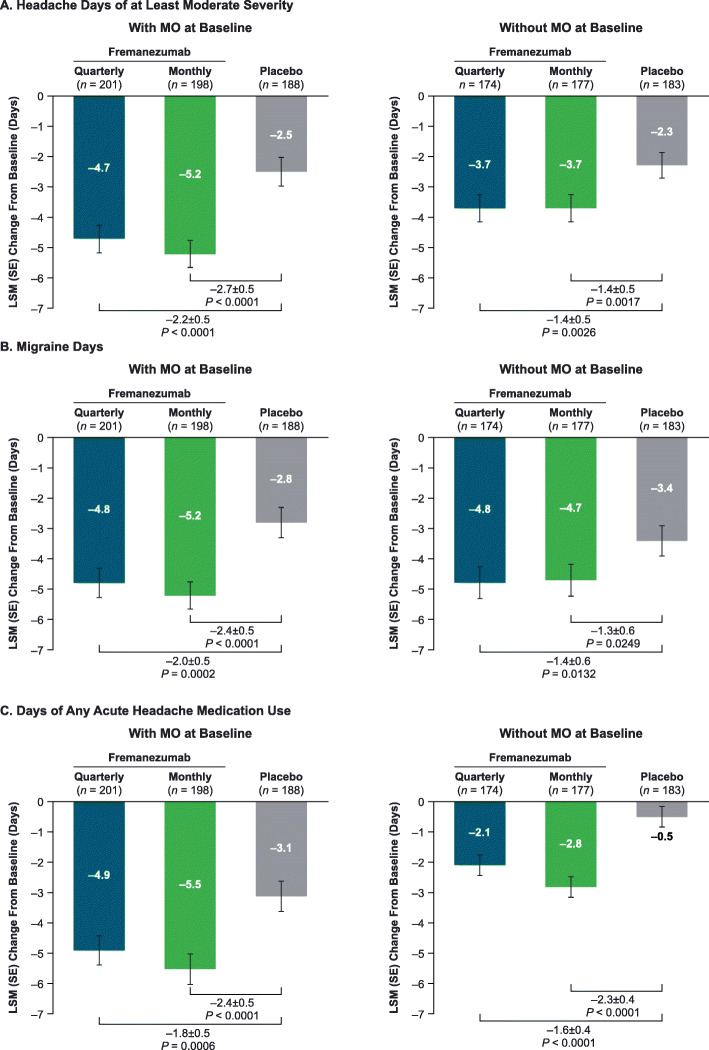
Change in days with (**a**) headache, (**b**) migraine, and (**c**) medication use in patients with CM by MO. Values shown are the mean changes from baseline in the monthly average number of (**a**) headache days of at least moderate severity, (**b**) migraine days, and (**c**) acute headache medication use during the 12-week treatment period with and without MO. CM, chronic migraine; LSM, least-squares mean; MO, medication overuse; SE, standard error

Among patients with MO at baseline, the placebo-adjusted LS mean (95% confidence interval [CI]) change from baseline in the monthly average number of headache days of at least moderate severity during the 12-week treatment period was significantly greater with fremanezumab quarterly (− 2.2 [− 3.1 to − 1.2]; *P* < 0.0001) and monthly (− 2.7 [− 3.7 to − 1.8]; *P* < 0.0001) (Fig. 1a). Similar results were seen in patients without MO at baseline (quarterly − 1.4 [− 2.3 to − 0.5], *P* = 0.0026; monthly − 1.4 [− 2.3 to − 0.6], *P* = 0.0017 vs placebo) (Fig. 1a). Fremanezumab-treated patients with MO had numerically greater reductions in the monthly average number of headache days of at least moderate severity than patients without MO and demonstrated a larger treatment effect over placebo than those with MO.

### Monthly average number of migraine days with and without MO

The placebo-adjusted LS mean (95% CI) reduction from baseline in monthly average number of migraine days in patients with MO at baseline was significantly greater with fremanezumab quarterly (− 2.0 [− 3.1 to − 1.0], *P =* 0.0002) and monthly (− 2.4 [− 3.5 to − 1.4], *P* < 0.0001) (Fig. 1b). Similar results were seen in patients without MO at baseline (quarterly − 1.4 [− 2.5 to − 0.3], *P =* 0.0132; monthly − 1.3 [− 2.4 to − 0.2], *P =* 0.0249 vs placebo]) (Fig. 1b). Numerically greater reductions in monthly average number of migraine days from baseline and a larger treatment difference over placebo were seen in fremanezumab-treated patients with MO compared with patients without MO.

### Acute headache medication use days with and without MO

Treatment with fremanezumab resulted in significantly greater reductions in the monthly average number of days of any acute headache medication use compared with placebo among patients with MO at baseline (LS mean difference [95% CI]: quarterly − 1.8 [− 2.9 to − 0.8], *P =* 0.0006; monthly − 2.4 [− 3.5 to − 1.4], *P* < 0.0001; Fig. 1c) and patients without MO at baseline (quarterly − 1.6 [− 2.4 to − 0.9], monthly − 2.3 [− 3.1 to − 1.6]; *P* < 0.0001 for both (Fig. 1c). Reductions from baseline in monthly average number of days with any acute headache medication use and treatment difference over placebo were numerically greater in fremanezumab-treated patients with MO than in patients without MO.

### ≥ 50% reduction in the monthly average number of headache days

**Fig. 2:**
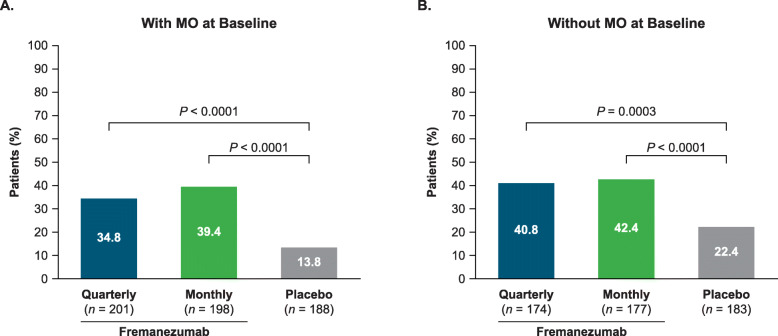
Proportion of patients with CM with ≥ 50% response (**a**) with MO and (**b**) without MO. A ≥ 50% response was defined as ≥ 50% reduction from baseline in the monthly average number of headache days of at least moderate severity over 12 weeks. CM, chronic migraine; MO, medication overuse

The proportion of patients with a ≥ 50% reduction in the monthly average number of headache days of at least moderate severity was significantly greater among fremanezumab quarterly and fremanezumab monthly groups compared with placebo, regardless of MO at baseline (with MO: quarterly 70/201 [34.8%, *P* < 0.0001], monthly 78/198 [39.4%, *P* < 0.0001] vs placebo 26/188 [13.8%]; without MO: quarterly 71/174 [40.8%, *P =* 0.0003], monthly 75/177 [42.4%, *P* < 0.0001] vs placebo 41/183 [22.4%, Fig. 2). The odds of achieving a ≥ 50% reduction (odds ratio [95% CI]) was greater with fremanezumab versus placebo in both patients with MO (quarterly 3.33 [2.01 to 5.52], monthly 4.08 [2.47 to 6.75]) and without MO (quarterly 2.37 [1.50 to 3.76], monthly 2.53 [1.60 to 4.00]).

### Patient-reported outcomes with and without MO

During the 12-week treatment period, the LS mean (standard error) change from baseline in HIT-6 scores was significantly greater with fremanezumab versus placebo, regardless of MO at baseline (with MO: quarterly LS mean − 6.0 [0.7], monthly − 6.9 [0.6] vs placebo − 4.5 [0.7]; Table 2; without MO: quarterly − 7.0 [0.7], monthly − 6.8 [0.6] vs placebo − 4.5 [0.6]; Table 2).

**Table 2:** Change from baseline in patient-reported outcome measures with and without MO

	CM With MO			CM Without MO		
	Fremanezumab			Fremanezumab		
	Quarterly (*n* = 201)	Monthly (*n* = 198)	Placebo (*n* = 188)	Quarterly (*n* = 174)	Monthly (*n* = 177)	Placebo (*n* = 188)
HIT-6						
LSM (SE)	−6.0 (0.7)	−6.9 (0.6)	−4.5 (0.7)	−7.0 (0.7)	− 6.8 (0.64)	−4.5 (0.6)
LSMD (SE)	− 1.5 (0.7)	−2.4 (0.7)		−2.4 (0.7)	− 2.3 (0.7)	
*P* value	0.0356	0.0009		0.0006	0.0012	
MSQoL, RFR						
LSM (SE)	19.6 (1.8)	21.4 (1.8)	14.7 (1.9)	21.9 (2.0)	21.6 (2.0)	14.5 (1.9)
LSMD (SE)	4.9 (2.0)	6.7 (2.0)		7.4 (2.1)	7.1 (2.1)	
*P* value	0.0142	0.0008		0.0005	0.0008	
MSQoL, RFP						
LSM (SE)	17.5 (1.7)	18.4 (1.6)	14.2 (1.7)	16.4 (1.7)	14.2 (1.7)	10.2 (1.7)
LSMD (SE)	3.2 (1.8)	4.2 (1.8)		6.2 (1.8)	3.9 (1.8)	
*P* value	0.0696	0.0200		0.0007	0.0290	
MSQoL, EF						
LSM (SE)	20.2 (2.0)	22.0 (1.9)	17.3 (2.0)	22.4 (2.1)	19.7 (2.1)	16.7 (2.1)
LSMD (SE)	2.9 (2.2)	4.7 (2.2)		5.7 (2.3)	3.0 (2.3)	
*P* value	0.1833	0.0305		0.0118	0.1873	
PHQ-9						
LSM (SE)	−2.8 (0.4)	−2.3 (0.4)	−2.4 (0.4)	−2.6 (0.4)	−2.3 (0.4)	−1.6 (0.4)
LSMD (SE)	−0.5 (0.4)	0.0 (0.4)		−1.0 (0.4)	−0.7 (0.4)	
*P* value	0.2679	0.9678		0.0155	0.0922	

*CM* Chronic migraine, *EF* Emotional function, *HIT-6* Six-item Headache Impact Test, *LSM* Least-squares mean, *LSMD* Least-squares mean difference, *MO* Medication overuse, *MSQoL* Migraine-Specific Quality of Life, *RFP* Role function–preventive, *RFR* Role function–restrictive, *PHQ-9* Patient Health Questionnaire-9, *SE* Standard error

LSMD was determined in comparison to placebo

Improvement in MSQoL domain scores was observed in patients with MO (RFR: quarterly 19.6 [1.8], monthly 21.4 [1.8] vs placebo 14.7 [1.9]; RFP: quarterly 17.5 [1.7], monthly 18.4 [1.6] vs placebo 14.2 [1.7]; EF: quarterly 20.2 [2.0], monthly 22.0 [1.9] vs placebo 17.3 [2.0]; Table 2). In the fremanezumab quarterly with MO group, the RFR domain score change from baseline compared with placebo was significant; the RFP and EF domain score changes were not significantly different from placebo (Table 2). All changes from baseline in MSQoL domain scores in the fremanezumab monthly group were significantly different compared with placebo (Table 2). Similarly, in patients without MO, improvements in MSQoL domain scores were observed (RFR: quarterly 21.9 [2.0], monthly 21.6 [2.0] vs placebo 14.5 [1.9]; RFP: quarterly 16.4 [1.7], monthly 14.2 [1.7] vs placebo 10.2 [1.7]; EF: quarterly 22.4 [2.1], monthly 19.7 [2.1] vs placebo 16.7 [2.1]; Table 2). All MSQoL domain scores in the fremanezumab quarterly without MO group were significantly different compared with placebo (Table 2). In the fremanezumab monthly without MO group, RFR and RFP domain score changes from baseline were significant compared with placebo; no significant difference in the EF domain score was observed (Table 2).

Reductions in PHQ-9 scores were observed, regardless of MO at baseline (with MO: quarterly − 2.8 [0.4], monthly − 2.3 [0.4] vs placebo − 2.4 [0.4]; without MO: quarterly − 2.6 [0.4], monthly − 2.3 [0.4] vs placebo − 1.6 [0.4]; Table 2). Numerically larger reductions were generally observed in the fremanezumab-treated groups compared with placebo; however, the only significant difference was observed in patients without MO in the fremanezumab quarterly group compared with placebo (Table 2).

### Reversion from MO to no MO

**Fig. 3:**
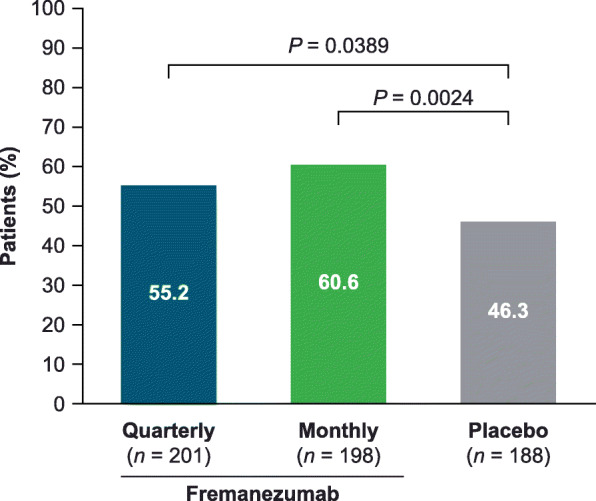
Proportion of patients with CM who reverted from MO to no MO during the 12-week treatment period. CM, chronic migraine; MO, medication overuse

Among CM patients with baseline MO, significantly greater proportions of patients treated with fremanezumab quarterly or monthly reverted to no MO during the 12-week treatment period (quarterly 111/201 [55.2%], *P =* 0.0389; monthly 120/198 [60.6%], *P =* 0.0024) than those who received placebo (87/188 [46.3%]) (Fig. 3). This effect was present by week 4 (quarterly 102/201 [50.7%], monthly 107/198 [54.0%] vs placebo 73/188 [38.8%]).

**Fig. 4:**
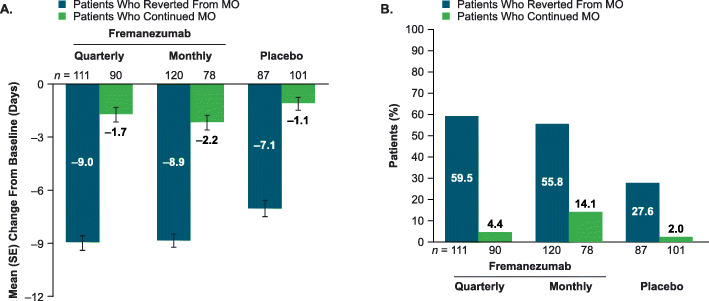
**(a)** Reduction in medication use and **(b)** ≥ 50% response in patients with CM by reversion to no MO. Values shown in part A are mean (SE) change from baseline over 12 weeks in the monthly average number of days of acute medication use in patients who reverted from MO to no MO (blue bars) and in those who did not revert from MO to no MO (green bars). Values shown in part B are the proportions of patients with a ≥ 50% response, defined as a ≥ 50% reduction in the monthly average number of headache days of at least moderate severity from baseline over 12 weeks, in patients who reverted from MO to no MO (blue bars) and in those who did not revert from MO to no MO (green bars). CM = chronic migraine; MO = medication overuse; SE = standard error

Similar baseline mean (standard deviation) monthly average number of days of acute headache medication use were observed across treatment arms within the subgroup of patients who reverted from MO (quarterly 16.6 [3.4], monthly 16.7 [3.6], placebo 16.6 [3.3]) and the subgroup of patients who continued MO (quarterly 19.9 [3.9], monthly 21.0 [4.5], placebo 19.5 [4.8]), though numerically greater numbers were observed among patients who continued MO. Among patients who reverted from MO at baseline, the reduction from baseline in the monthly average number of days of acute headache medication use was − 9.0 (0.4) with fremanezumab quarterly, − 8.9 (0.4) with fremanezumab monthly, and − 7.1 (0.5) with placebo (Fig. 4a). In comparison, patients with continued MO experienced numerically smaller reductions in the monthly average number of days of acute medication use in both the fremanezumab-treated groups (quarterly − 1.7 [0.4], monthly: − 2.2 [0.4]) and the placebo group (− 1.1 [0.4]; Fig. 4a).

Furthermore, more than half of the patients who reverted from MO achieved a ≥ 50% reduction in the monthly average number of headache days of at least moderate severity after treatment with fremanezumab quarterly (66/111 [59.5%]) or monthly (67/120 [55.8%]); of those treated with placebo, 27.6% (24/87) achieved a ≥ 50% reduction (Fig. 4b). Among patients who continued to experience MO, the proportion of patients with a ≥ 50% reduction in the monthly average number of headache days of at least moderate severity was 4.4% (4/90) with fremanezumab quarterly, 14.1% (11/78) with fremanezumab monthly, and 2.0% (2/101) with placebo (Fig. 4b).

## Discussion

These post hoc analyses demonstrated that fremanezumab, compared with placebo, significantly reduced the monthly average number of headache days of at least moderate severity and led to a significantly greater proportion of patients who had a clinically meaningful ≥ 50% response rate, independently of the presence of MO in patients with CM. Similar reductions were also seen for migraine days. In patients with baseline MO, significantly more fremanezumab-treated patients reverted to no MO during the 12-week treatment period compared with those who received placebo. Significant reductions in days using acute headache medication were also observed in patients with and without MO at baseline, with the greatest numerical reductions noted in those who reverted from MO to no MO during the 12-week treatment period.

Migraine preventive therapy is recommended for patients with frequent and chronic migraine, but the effectiveness of preventive medications was believed to be affected by MO [6]. Topiramate, a migraine preventive treatment, significantly reduced migraine days in patients with CM, but failed to reach significance in a post hoc analysis of patients with CM and MO, possibly due to a small sample size [21]. In one study of patients with MO who were unresponsive to preventive therapy, withdrawal of the overused medication increased the efficacy of preventive therapy [22]. However, in our study, more patients treated with fremanezumab had a ≥ 50% reduction in the monthly number of headache days of at least moderate severity, independent of baseline MO. This strongly suggests that MO has minimal, if any, impact on the efficacy of fremanezumab.

Notable differences were observed between patients with and without MO at baseline, including several differences in disease characteristics. By definition, monthly number of days using acute headache medication differed between subgroups. The defining feature of the subgroups may also underlie the differences in monthly number of headache days of at least moderate severity and migraine days, because a day with acute migraine-specific medication use to treat a headache of any severity or duration was included in the count for these variables. Disease characteristics that did not notably differ at baseline included levels of disability, quality of life, and depression as measured by HIT-6, MSQoL domain, and PHQ-9 scores, respectively. Previous studies have shown that patients with chronic daily headache (CDH; an umbrella term that encompasses CM) and MO have more disability and quality-of-life impairment compared with patients with CDH without MO; however, because these studies looked at MO in a heterogenous group of headache disorders, they may not be generalizable to patients with CM [1]. Similarly to CM, medication overuse headache, a secondary headache attributable to MO, is associated with psychiatric comorbidities such as depression, although whether it could be a cause or an effect is not known [23, 24]. Baseline characteristics in this study suggest that MO does not increase the severity of depression in patients with CM. The impact of fremanezumab on disability, quality of life, and depression was positive in patients with and without MO, with significant differences from placebo observed for HIT-6 and MSQoL domain scores. PHQ-9 scores were reduced from baseline; however, only the reduction among patients without MO in the fremanezumab quarterly group reached statistical significance. A greater difference over placebo may not have been observed due to the majority of patients (69.1% to 79.2%) already having PHQ-9 scores in the no to minimal depression range (R.B. Lipton, unpublished data, 2019), creating a floor effect. In a separate subgroup analysis of patients in this population with moderate to severe depression, reductions in mean PHQ-9 scores were numerically greater, although not statistically significant, potentially due to the modest sample size (R.B. Lipton, unpublished data, 2019).

In this study, patients with MO saw improvements in the measured outcomes without undergoing withdrawal therapy or so-called “detoxification,” which is often believed to be important in managing MO [2]. Several strategies are available for detoxification, including early abrupt withdrawal, restricted intake, discontinuation therapy with rescue medication (different analgesic than the overused medication), or intravenous hydration [2, 3, 25]. During detoxification, most patients experience an initial worsening of headaches and additional withdrawal symptoms such as nausea, vomiting, hypotension, tachycardia, sleep disturbances, restlessness, and anxiety, which typically last up to 10 days, but may persist for up to 4 weeks [3, 26, 27]. Studies have demonstrated variable rates of success in discontinuing medications, with percentages of patients achieving detoxification ranging from 57% to 100% [28]. Even after successful discontinuation of overused medication, 17% to 43% of patients relapse back to MO just 1 year after detoxification [28]. Our results suggest that patients treated with fremanezumab do not need to discontinue overused drugs to experience the benefit of preventive treatment and achieve reduced acute medication use. Fremanezumab treatment resulted in 58% of patients reverting from MO to no MO, compared with 46% for patients given placebo, suggesting that patients receiving fremanezumab may achieve reversion from MO, avoid detoxification and its associated symptoms, and still achieve fewer headache days and migraine days. Together, this suggests the potential for a paradigm shift to an effective and more humane approach for treating CM with MO: prevent first and detoxify later if necessary.

These findings have certain limitations. These subgroup analyses were not prespecified in the HALO CM protocol; however, the measures evaluated were generally consistent with the observed effects in the FAS population, and all data points in these subgroups were collected a priori. In addition, this study did not consider the intensity of MO, differentiate between the types or combination of medications taken, nor did it consider the presence and severity of comborbid psychiatric disorders that may contribute to MO, such as anxiety or cephalophobia. Future studies to determine whether the type of overused medication impacts outcomes would be beneficial. Due to exclusion criteria, patients using opioids or barbiturates on more than 4 days per month were not included in this trial; therefore, inferences cannot be drawn about patients overusing these drugs. Furthermore, one cannot rule out that the routine observation by doctors over the course of the trial may have contributed to some of the observed reductions in MO; however, placebo-treated patients were observed in a similar manner with smaller improvements. The current understanding of the pathophysiology of MO also supports the role of fremanezumab in reducing MO. Increased CGRP expression and release is hypothesized to play a role in the development of the increased sensitivity to pain perception that MO fosters [29, 30]. This sensitization may be reversed by blocking CGRP with fremanezumab. In triptan- or morphine-sensitized rats, a single administration of fremanezumab was able to prevent cutaneous allodynia resulting from triggers associated with migraine attacks [31]. Lastly, headache data were captured using a patient self-reported diary, which can vary in terms of content recorded, reading and writing skill, and compliance.

## Conclusion

Results from these post hoc analyses demonstrated that fremanezumab was efficacious in the preventive treatment of patients with CM regardless of the presence of MO and increased the likelihood of reversion from medication overuse to no medication overuse.

## Supplementary information

**Additional file 1: Table S1.** HALO CM key inclusion and exclusion criteria.

## Data Availability

Anonymized data, as described in this manuscript, will be shared upon request from any qualified investigator by the author investigators or Teva Pharmaceutical Industries, Ltd.

## Abbreviations

CDH: Chronic daily headache
CGRP: Calcitonin gene-related peptide
CI: Confidence interval
CM: Chronic migraine
EF: Emotional function
FAS: Full analysis set
HD: Headache day of at least moderate severity
HIT-6: Six-item Headache Impact Test
ICHD-3: International Classification of Headache Disorders, third edition
LS: Least-square
MO: Medication overuse
MSQoL: Migraine-Specific Quality of Life
PHQ-9: Nine-item Patient Health Questionnaire
RFP: Role function–preventive
RFR: Role function–restrictive
SD: Standard deviation
SE: Standard error
