# Supplementary material for: The impact of fremanezumab on medication overuse in patients with chronic migraine: subgroup analysis of the HALO CM study
Source: J Headache Pain. 2020 Sep 21;21(1):114. doi: 10.1186/s10194-020-01173-8 (PMC7507645; doi:10.1186/s10194-020-01173-8)
Supplement: Supplementary file 1 — Additional file 1: Table S1. HALO CM key inclusion and exclusion criteria. [file 10194_2020_1173_MOESM1_ESM.docx]

**Additional file 1.**

**Table:** **HALO CM key inclusion and exclusion criteria.**

| **Key Inclusion Criteria** | **Key Exclusion Criteria** |
| --- | --- |
| - 18–70 years of age - History of migraine (International Classification of Headache Disorders, third edition, beta version [ICHD-3 beta]) for ≥12 months prior to screening - Prospectively confirmed CM during the 28-day pre-treatment baseline period   - Headache on ≥15 days   - ≥8 days fulfilling ICHD-3 beta criteria for migraine, probable migraine, or use of triptan or ergot medications - A subset of patients was allowed use of one preventive migraine medication if the dosage was stable for ≥2 months before the pre-treatment period | - Use of onabotulinumtoxinA in the 4 months before screening - Use of opioids or barbiturates on >4 days per month during the pre-treatment period - Use of interventions or devices for migraine in the 2 months before screening - Previous failure (lack of efficacy after adequate therapeutic trial) in ≥2 of the following medication clusters after ≥3 months of treatment for EM or CM:   - Divalproex sodium and sodium valproate   - Flunarizine and pizotifen   - Amitriptyline, nortriptyline, venlafaxine, and duloxetine   - Atenolol, nadolol, metoprolol, propranolol, and timolol |

CM = chronic migraine; EM = episodic migraine; ICHD-3 beta = International Classification of Headache Disorders, 3rd edition beta.
